# Supplementary material for: Prevalence of clinical signs of possible serious bacterial infection and mortality associated with them from population-based surveillance of young infants from birth to 2 months of age
Source: PLoS One. 2021 Feb 24;16(2):e0247457. doi: 10.1371/journal.pone.0247457 (PMC7904202; doi:10.1371/journal.pone.0247457)
Supplement: Attachment — Submitted filename: 2021-1-13_Rebuttal letter_PONE-D-20-16849.docx [file pone.0247457.s001.docx]

**Point by point response to reviewers’ comments**

**Manuscript no:** PONE-D-20-16849

**Title:** Prevalence of clinical signs of possible serious bacterial infection and mortality associated with them from a population-based surveillance of young infants from birth to 2 months of age

**Reviewer #1:**

I read the manuscript in 1 hour. I'm enthusiastic about the manuscript. I find it well wrote and with important topic. Below my suggestions

**Comment # 1.1**

Introduction: well done. underline better also the role of maternal caesarian section infection and how it is important also for maternal and neonatal outcome especially in low setting (see and cite this paper from Sierra Leone Di Gennaro F, Maternal caesarean section infection (MACSI) in Sierra Leone: a case-control study. Epidemiol Infect. 2020 Feb 27;148:e40. doi: 10.1017/S0950268820000370. PMID: 32102721; PMCID: PMC7058652.) and how maternal and neonatal outcomes are strongly related.

**Authors’ response**

Thank you. We have added a sentence. ‘ Maternal sepsis during pregnancy, labour and post-partum and post caesarean section infections also result in neonatal deaths in low resource settings.’^[1]^ in the ‘Introduction’. See lines 70-71

**Comment #1.2**

Methods well wrote

**Authors’ response**

Thank you.

**Comment #1.3**

Results: no comment

**Authors’ response**

Thank you.

**Comment# 1.4**

Discussion: underline the lack of microbiological test for the identification of responsible germs and a better antibiotic therapy and identification of resistance. I believe there is a need to advocate on the development of microbiology in these contexts. Underline also the need to develop an Intensive Care unit for maternal and neonatal (see and cite this experience: Epidemiology, Outcomes, and Risk Factors for Mortality in Critically Ill Women Admitted to an Obstetric High-Dependency Unit in Sierra Leone. Am J Trop Med Hyg. 2020 Nov;103(5):2142-2148. doi: 10.4269/ajtmh.20-0623. PMID: 32840199; PMCID: PMC7646769. and Cost-Utility of Intermediate Obstetric Critical Care in a Resource-Limited Setting: A Value-Based Analysis. Ann Glob Health. 2020 Jul 20;86(1):82. doi: 10.5334/aogh.2907. PMID: 32742940; PMCID: PMC7380057.)

**Authors’ response**

We have added, ‘Fourth, high early neonatal mortality rate is also linked with complications in pregnancy, labour and post-partum, so there it is essential to timely identify high-risk pregnancies and manage them in suitable high dependency and critical care settings in low and middle-income countries, which is challenging.’^[2]^ in ‘Discussion’ – see lines 286-289.

**Comment #1.5**

Also highlighted the need for doctors to train themselves on the topics of antibiotic therapy, antimicrobial stewardship because the role of sepsis in neonatal and maternal deaths is very high (doi: 10.1016/j.jgar.2020.08.022. Epub ahead of print. PMID: 32971291.)

**Authors’ response**

We have added, ‘Finally, prompt identification and diagnosis of neonatal sepsis require antibiotics and supportive treatment. Inappropriate antibiotic use contributes to antimicrobial resistance, which is quite widespread even in the high-income countries and is one of the greatest public health challenges of the 21st century.^[3]^ Antibiotic stewardship is essential and health professionals treating sepsis should have the knowledge and follow the recommended antibiotic practices in light of the increasing antimicrobial resistance and avoid using unsuitable treatments.’^[4]^ In ‘Discussion’ – see lines 289-294.

**Reviewer #2:**

**Comment #2.1**

Hypothermia is defined in your study as < 35.5, and but the WHO its publication as well as in US NWN nurseries and defined by AAP, hypothermia in neonates is < 36.5 (few centers refer to 36.3). Please explain why you use lower temp Also, if you use WHO definition as listed below, that can completely change incidence of morbidity among the population you visited: <https://apps.who.int/iris/bitstream/handle/10665/60042/WHO_FHE_MSM_93.2.pdf;jsessionid=08EF4625562B4D043C61B201EEFE6C26?sequence=1>

* keep in your mind: In newborns, the rectal temperature was equal to the axillary temperature plus 0.2 degrees C for each week of age up to 5 weeks. DOI: 10.1001/archpedi.1996.02170260078013

**Authors’ response**

Thank you so much for your comment. The above-cited WHO document was published in 1993. We have used an updated WHO IMCI chart booklet published in 2014 to define hypothermia, which was defined as an axillary temperature of less than 35.5^o^C (reference 8 in the manuscript) - see line 95.^[5]^

**Comment #2.2**

for Signs of Systemic Infection: Our authors did not mention color change:

- Jaundice other than physiologic one (prolonged and deep color)
- Cyanosis (central)
- Skin rash
- excessive losing Body weight compared to birth weight, which is a sign of underlying pathology vs. malnutrition.

Please explain.

**Authors’ response**

Community health workers (CHW) are non-medical community personnel who are usually trained for a few weeks. Using WHO training materials, we trained the CHWs in caring for newborns and identifying sick young infants, especially those with an infection.^5,^^[6]^ (references 8 and 30 in the manuscript) A minimal number of signs were included in the training materials, which were shown to have a combined high sensitivity and specificity^[7]^ (reference 7 in the manuscript) to identify sick young infants and included in the young infant component of the WHO/UNICEF IMCI chart booklet.^5^ The signs mentioned by the reviewer were not part of their training, except for ‘yellow soles’ to identify severe jaundice, which was included in the training materials. We didn’t report data on jaundice because it was not included as part of the WHO definition of ‘possible serious bacterial infection’.

1. WHO Global Maternal Sepsis Study (GLOSS) Research Group. Frequency and management of maternal infection in health facilities in 52 countries (GLOSS): a 1-week inception cohort study. Lancet Glob Health. 2020;8:e661-e671. doi: 10.1016/S2214-109X(20)30109-1.

   Chu K, Maine R, Trelles M. Cesarean section surgical site infections in sub-Saharan Africa: a multi-country study from Medecins Sans Frontieres. World J Surg. 2015;39:350-5. doi: 10.1007/s00268-014-2840-4.

   Di Gennaro F, Marotta C, Pisani L, Veronese N, Pisani V, Lippolis V, Pellizer G, Pizzol D, Tognon F, Bavaro DF, Oliva F, Ponte S, Nanka Bruce P, Monno L, Saracino A, Koroma MM, Putoto G. Maternal caesarean section infection (MACSI) in Sierra Leone: a case-control study. Epidemiol Infect. 2020;148:e40. doi: 10.1017/S0950268820000370.
2. Vasco M, Pandya S, Van Dyk D, Bishop DG, Wise R, Dyer RA. Maternal critical care in resource-limited settings. Narrative review. Int J Obstet Anesth. 2019 Feb;37:86-95. doi: 10.1016/j.ijoa.2018.09.010.

   Marotta C, Pisani L, Di Gennaro F, Cavallin F, Bah S, Pisani V, Haniffa R, Beane A, Trevisanuto D, Hanciles E, Schultz MJ, Koroma MM, Putoto G. Epidemiology, Outcomes, and Risk Factors for Mortality in Critically Ill Women Admitted to an Obstetric High-Dependency Unit in Sierra Leone. Am J Trop Med Hyg. 2020 Nov;103(5):2142-2148. doi: 10.4269/ajtmh.20-0623.

   Marotta C, Di Gennaro F, Pisani L, Pisani V, Senesie J, Bah S, Koroma MM, Caracciolo C, Putoto G, Amatucci F, Borgonovi E. Cost-Utility of Intermediate Obstetric Critical Care in a Resource-Limited Setting: A Value-Based Analysis. Ann Glob Health. 2020 Jul 20;86(1):82. doi: 10.5334/aogh.2907.
3. Cassini A, Högberg LD, Plachouras D, Quattrocchi A, Hoxha A, Simonsen GS, Colomb-Cotinat M, Kretzschmar ME, Devleesschauwer B, Cecchini M, Ouakrim DA, Oliveira TC, Struelens MJ, Suetens C, Monnet DL; Burden of AMR Collaborative Group. Attributable deaths and disability-adjusted life-years caused by infections with antibiotic-resistant bacteria in the EU and the European Economic Area in 2015: a population-level modelling analysis. Lancet Infect Dis. 2019;19:56-66. doi: 10.1016/S1473-3099(18)30605-4.
4. Di Gennaro F, Marotta C, Amicone M, et al. Italian young doctors' knowledge, attitudes and practices on antibiotic use and resistance: A national cross-sectional survey. JGAR. 2020;23:167-173. DOI: 10.1016/j.jgar.2020.08.022.

   Pulcini C, Williams F, Molinari N, Davey P, Nathwani D. Junior doctors' knowledge and perceptions of antibiotic resistance and prescribing: a survey in France and Scotland. Clin Microbiol Infect. 2011 Jan;17(1):80-7. doi: 10.1111/j.1469-0691.2010.03179.x.
5. World Health Organization. Integrated Management of Childhood Illness (IMCI): Chart Booklet. Geneva, Switzerland: WHO, 2014. <https://www.who.int/maternal_child_adolescent/documents/IMCI_chartbooklet/en/>.
6. World Health Organization, UNICEF. Integrated Management of Childhood Illness: caring for newborns and children in the community. Switzerland: WHO & UNICEF, 2011. <https://apps.who.int/iris/handle/10665/44398>
7. Young Infants Clinical Signs Study Group. Clinical signs that predict severe illness in children under age 2 months: a multicentre study. Lancet. 2008;371(9607):135-42.
